# Effective treatment of sarcoptic mange in an alpaca (*Vicugna pacos*) using fluralaner: a case report

**DOI:** 10.1007/s11259-024-10316-0

**Published:** 2024-01-27

**Authors:** Giulia Sala, Alessia Libera Gazzonis, Davide Pravettoni, Alessandra Cafiso, Guido Grilli, Vincenzo Ferrulli, Antonio Boccardo, Federica Di Cesare, Laura Filippone Pavesi, Sergio Zanzani

**Affiliations:** 1 Department of Veterinary Sciences, University of Pisa, via Livornese s.n.c, San Piero a Grado, 56122 Italy; 2 Department of Veterinary Medicine and Animal Sciences, Università degli Studi di Milano, via dell’Università 6, Lodi, 26900 Italy

**Keywords:** South American camelids, Alpaca, Sarcoptic mange, Fluralaner, *Sarcoptes scabiei*

## Abstract

South American Camelids, including alpacas, have gained popularity in Europe as pets and prized wool sources. Skin health concerns, particularly mite infestations, have emerged as a notable problem in these animals. Sarcoptic mange can lead to severe itching, papules, and chronic symptoms such as alopecia, crusts, and emaciation if left untreated. This case report documents a 2-year-old female alpaca suffering from sarcoptic mange. Despite initial treatment with ivermectin, its condition worsened, leading to severe weight loss, abortion, and a continued presence of mites. Considering the lack of effective treatments for sarcoptic mange in alpacas and the unavailability of registered drugs for this species in Italy, fluralaner, a drug previously used in other animal species, has been administered orally at a dosage of 5 mg/kg. Within a week after the treatment with fluralaner, the patient exhibited significant improvement, including the resolution of itching, healing of skin lesions, and an increase in appetite. Follow-up skin scrapings confirmed the absence of mites, and the patient’s condition continued to improve. Fluralaner demonstrated to be a highly effective and fast-acting treatment for sarcoptic mange in alpacas, offering potential economic benefits attributed to its single-dose administration.

## Introduction

South American camelids are highly versatile mammals native to South America, primarily raised for their meat, hides, and prized wool. Over the past few decades, their popularity has surged in Europe, where they are now bred as pets or valuable sources of wool (Bates et al. 2001; Beck 2020). Among health concerns, mite infestations have emerged as a notable problem. Three genera of mites – *Sarcoptes*, *Chorioptes*, and *Psoroptes* – have been documented as capable of infestating llamas and alpacas in the Americas and Europe (Johnson 1994; Curtis et al. 2001; Geurden et al. 2003; Gomez-Puerta et al. 2013; Beck 2020; Deak et al. 2021; Castilla-Castaño et al. 2021). Sarcoptic mange can cause severe skin disorders in South American camelids (Beck 2020; Gomez-Puerta et al. 2013; Castilla-Castaño et al. 2021). This type of mange leads to severe itching and small papules initially on the head/face and/or legs, but it can spread throughout the entire body in severe infestations. If left untreated, the acute stage of the disease can progress into a chronic form characterized by alopecia, crusts, excessive keratinization, an increase in connective tissue, and lichenification (Bornstein and de Verdier 2010; Gomez-Puerta et al. 2013). This could result in an unfavorable energy imbalance, gradual emaciation, and reduced feed intake (Bornstein and de Verdier 2010). Sarcoptic mange spreads rapidly within herds, primarily through direct and indirect contact (Ferreyra et al. 2022). Furthermore, it is a significant zoonotic disease (Moroni et al. 2022).

The treatment of sarcoptic mange in alpaca (*Vicugna pacos*) has proven to be particularly challenging (Hunter et al. 2004; D’Alterio et al. 2005; Borgsteede et al. 2006; Twomey et al. 2009; Pollock et al. 2017; Beck 2020). The absence of lanolin (wool grease), typical of camelids, limits the use of topical products that are effective for other species, such as sheep and cattle (Hunter et al. 2004; Bornstein et al. 2010). Furthermore, registered medications specifically for camelids are not available in Italy and are rare elsewhere. Therefore, treatment in alpacas is often performed off-label. Effective treatments for sarcoptic mange in alpacas have been achieved using macrocyclic lactones either alone or in combination with amitraz (Deak et al. 2021; Beck 2020; Lau et al. 2007; Twomey et al. 2009; Castilla-Castaño et al. 2021). However, macrocyclic lactones alone has yielded conflicting results in the literature (Borgsteede et al. 2006; Pollock et al. 2017). Moreover, in Italy, products containing amitraz are no longer available. In other species, mange treatment has achieved effective results using isoxazolines. For instance, the molecule fluralaner has proven effective against different mite species in companion animals (Taenzler et al. 2017; Chiummo et al. 2020; d’Ovidio and Santoro 2021) and recently in infestations by *Dermanyssus gallinae* in hens (Petersen et al. 2021) and in psoroptic mange in bighorn sheep (Hering 2020). This study describes the therapeutic success of using fluralaner in the treatment of sarcoptic mange in an alpaca following an unresponsive therapy with ivermectin.

## Materials and methods

### Case included in the study and clinical signs

The study was carried out on a 2-year-old pregnant female alpaca, part of a herd of 15 female alpacas and 2 crias raised by a private breeder for their valuable wool. These animals were kept together in an outdoor paddock with access to an indoor shelter.

In June 2022, the alpaca was temporarily relocated to another breeding facility for mating and returned to the herd in September 2022. The herd at the breeding facility was composed of 6 alpacas. Upon its return, the alpaca began scratching itself, and the owner observed the development of alopecia and crusts on its head. A veterinarian practitioner suspected mange based on clinical signs and treated alpaca with ivermectin (0.2 mg/kg; Ivomec, Boehringer Ingelheim Animal Health, Italy). No other animals in the herd showed clinical signs of mange. The alpaca not improved clinical condition and then was referred to the Ruminant and Swine Clinic at the Department of Veterinary Medicine and Animal Science, University of Milan (VTH), and admitted for isolation due to the potential zoonotic nature of the condition.

**Fig. 1:**
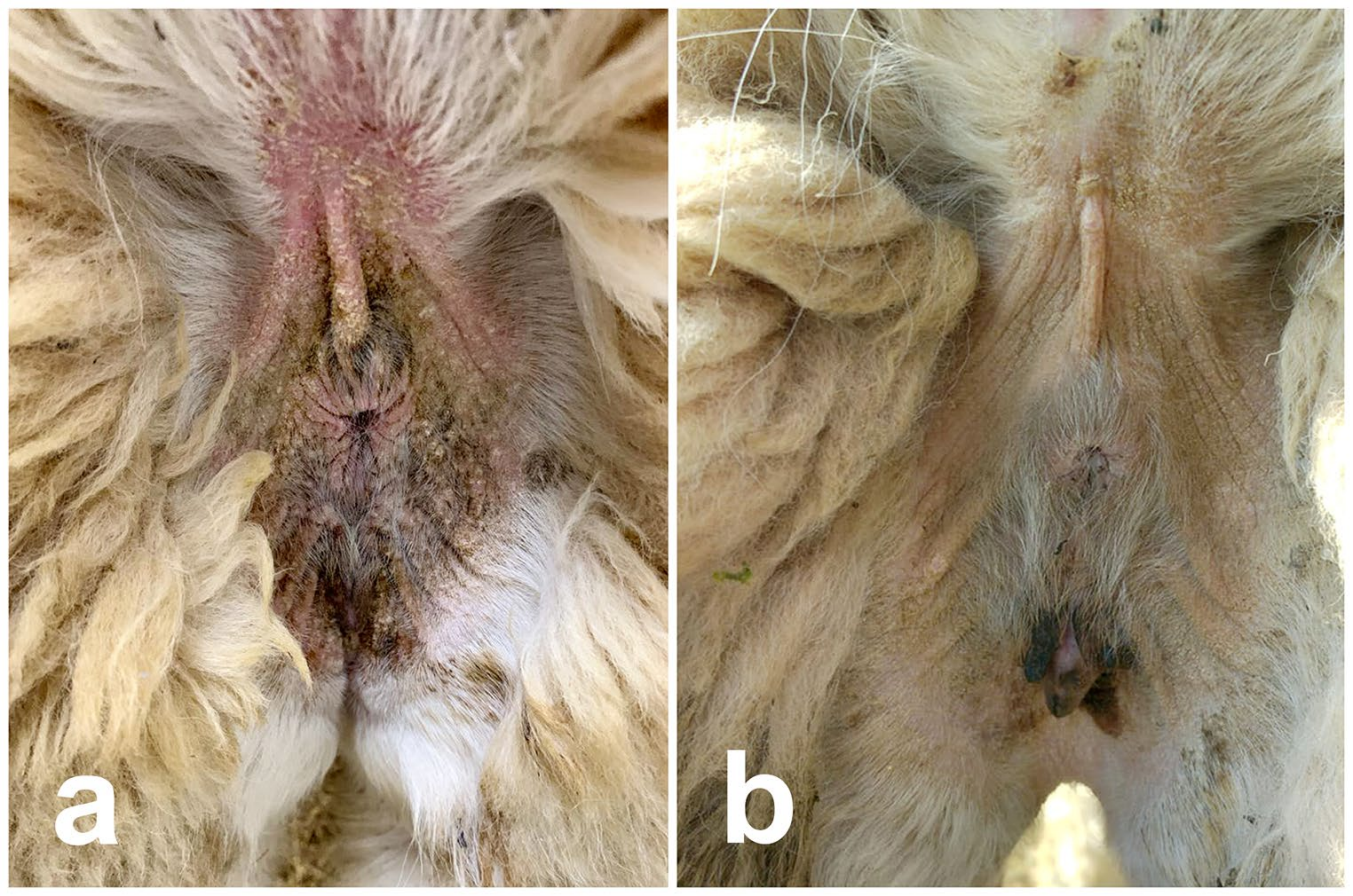
Improvement of the clinical lesion after treatment with fluralaner (5 mg/kg oral) of the perineal region. Picture (**a**) was taken on 5th December 2022, before the treatment with fluralaner, while picture (**b**) was taken after 90 days after treatment (4th March 2023)

At the time of admission on November 5, 2022, the animal was alert, with normal cardiac and respiratory parameters, and its major organ functions were well-maintained. The weight was 65 kg, and the body condition score (BCS) was 2.5 out of 5 (Van Saun 2003). Examination of the skin revealed alopecic areas in the perineal region, inner thigh, ventral abdomen, axillary region, neck, and head, exhibiting crusts, hyperkeratosis, and yellowish exudate compatible with bacterial superinfection (Fig. 1). The animal also displayed intense itching. After 3 weeks of hospitalization, the animal also experienced an abortion, and the weight decreased from 65 kg to 56 kg, with a BCS of 1.5.

### Parasitology and DNA analysis

**Fig. 2:**
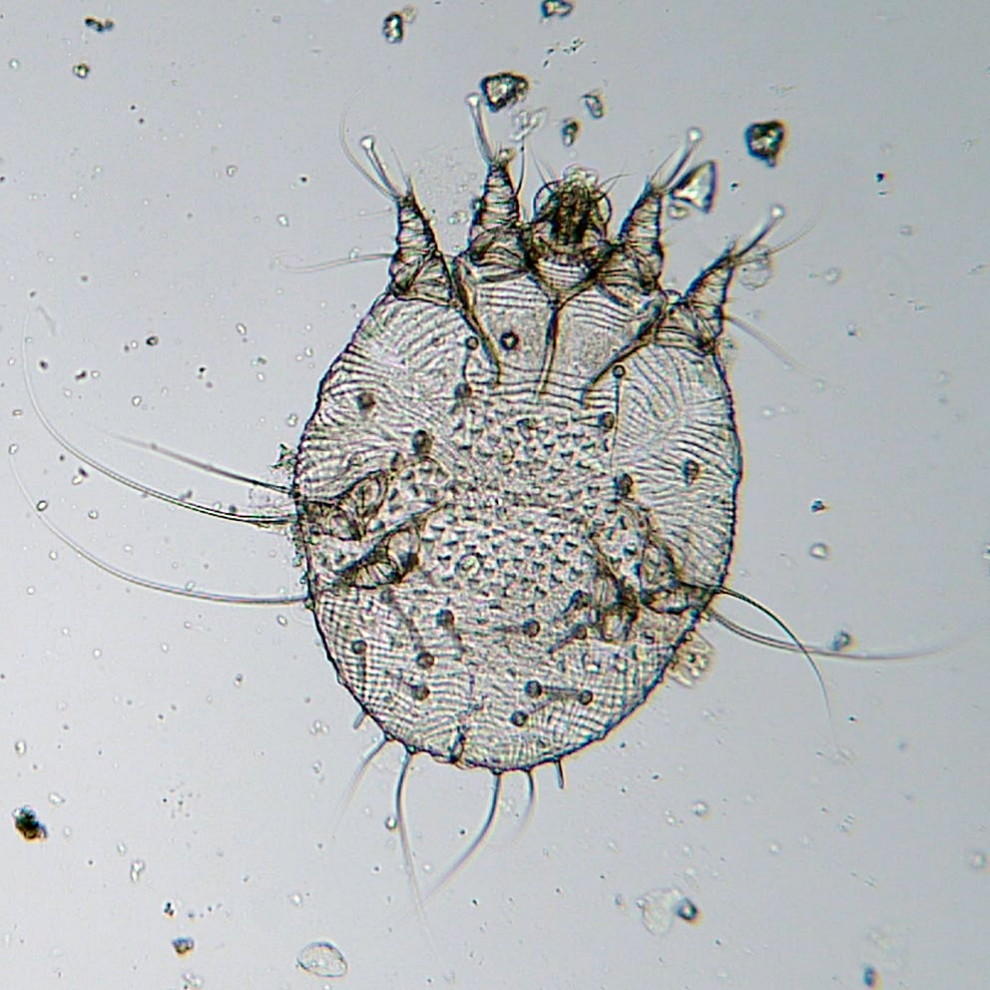
An adult female of *Sarcoptes scabiei* isolated by skin scraping from the described case. Magnification: 100x

**Fig. 3:**
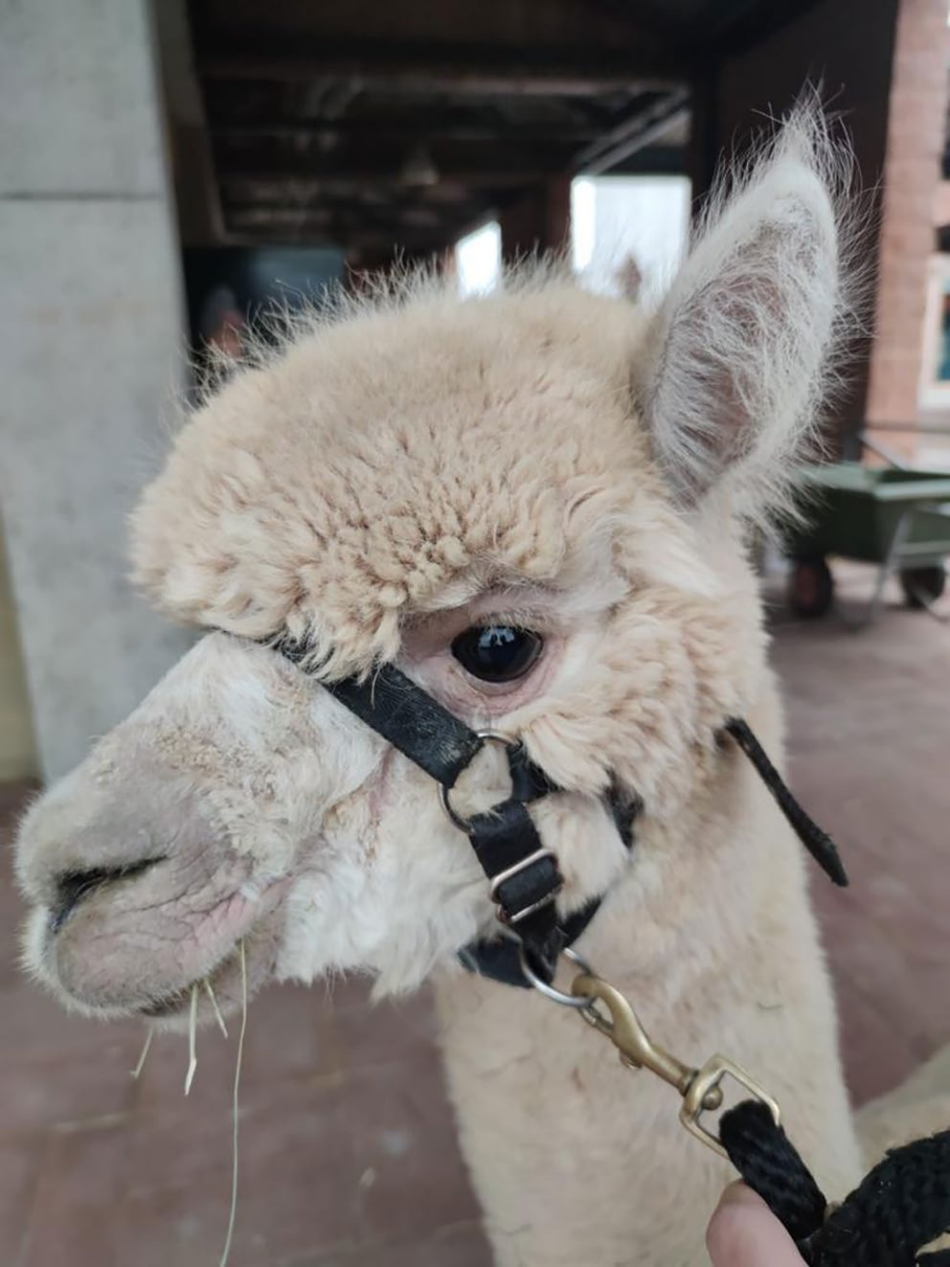
Improvement of clinical lesion of the head 15 days after treatment with fluralaner (5 mg/kg oral, 20th December 2022)

During first examination at VTH, deep skin scrapings were performed at the edge of each lesion from the head, thigh, and perineal region. In the laboratory, the material was transferred into a Petri dish containing 10% KOH solution and left for digestion for at least 2–3 h at room temperature, then inspected under a stereomicroscope (Fig. 2). Several mites were found and morphologically identified as *S. scabiei* (Fig. 3) (Deplazes et al. 2016).

To molecularly confirm the identity of the mites, a portion of the skin scrapings was subjected to DNA extraction using the DNeasy Blood and Tissue kit (Qiagen, Hilden, Germany) according to the manufacturer’s instructions. A ~ 400 bp fragment of the cytochrome *c* oxidase I (*coxI*) gene was PCR-amplified based on published protocols (Fournier et al. 1994; Zhou et al. 2022) and the purified amplicons were bidirectionally Sanger sequenced, and the obtained sequence (accession OR668927) was submitted to BLAST analyses, resulting in a 99–100% match with *S. scabiei* sequences available in GenBank.

### Therapeutic approach and pharmacological treatment

At VTH, a second subcutaneous administration of ivermectin at a dose of 0.2 mg/kg (Ivomec, Boehringer Ingelheim Animal Health, Italy) were performed (Twomey et al. 2009). Additionally, weekly cleansing with chlorhexidine shampoo and systemic treatment with procaine penicillin at 20,000 IU/kg, administered subcutaneously twice daily for 7 days (Prontocill, ATI, Italy) (D’Alterio 2006) was initiated to address the established skin superinfection. After 3 weeks of hospitalization, given the lack of efficacy of treatment with macrocyclic lactones, the unavailability of amitraz-containing medications in Italy, and considering the worsening clinical condition of the animal, with the informed consent of the owners, another registered drug in Italy for the treatment of sarcoptic mange in different species was chosen (Chiummo et al. 2020). In particular, fluralaner was selected, which had previously been successfully used for the treatment of psoroptic mange in another polygastric species, the bighorn sheep (Hering 2020), at an effective lower dosage of 5 mg/kg (Exzolt, MSD Animal Health S.r.l., Italy). The choice to use the oral solution was made based on the results observed in the mentioned study, where the oral preparation of fluralaner was more effective than topical administration (Hering 2020).

## Results

After one week of hospitalization, the bacterial superinfection of the affected skin areas was resolved. However, the animal’s condition continued to deteriorate, with the crusts on the face extending to the left eyelid and lips, and a decreased appetite and weight. A parasitological examination of the skin scraping conducted after 15 days performed after the second ivermectin treatment still indicated the presence of *S. scabiei*, and, considering the worsening of the animal’s condition, it was decided to change the pharmacological treatment, using fluralaner.

One week after treatment with fluralaner, the itching had disappeared, the animal had reopened its left eye, lip lesions had improved, and the animal resumed eating with appetite (Fig. 2). A skin scraping performed 15 days after fluralaner administration still yielded a positive result. Still, the animal was discharged and placed in a dedicated stall due to the clinical improvement.

**Fig. 4:**
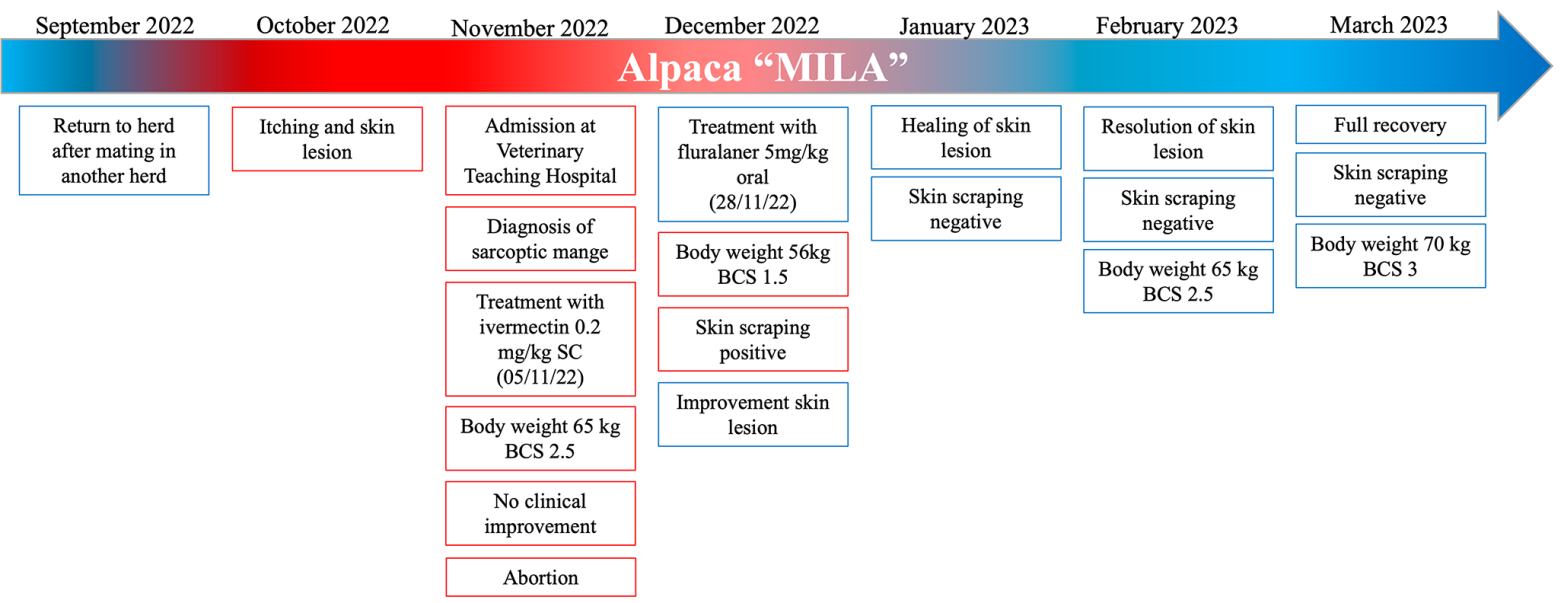
Timeline of the sarcoptic mange clinical case treated with fluralaner described in this study

One month after treatment with fluralaner, the skin lesions were healing, and the skin scraping yielded negative results. The animal was monitored for an additional 2 months, with skin scrapings conducted every 15 days; all of which returned negative results. Furthermore, the skin lesions had completely healed. The alopecic areas were no longer present (Fig. 1). Additionally, the animal had regained weight: 56 kg (BCS 1.5) at the time of fluralaner administration, 62 kg (BCS 2.5) one month after treatment, and 70 kg (BCS 3) at the last checkup conducted three months after the treatment (Fig. 4).

**Fig. 5:**
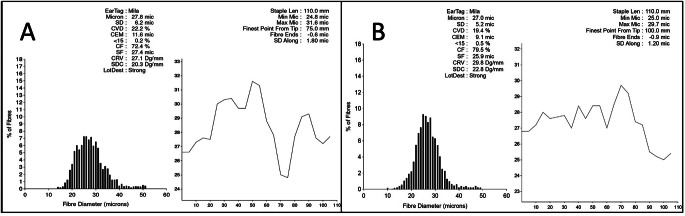
Figure **A** reported the result of the Mila fiber test before the episode of mange (May 2022), while Figure **B** reported the result after the resolution of mange (May 2023). The quality of the fiber was found to be comparable between the two years, regardless of the episode of sarcoptic mange

Seven months after treatment, the patient was sheared, and the fiber was subjected to analysis by an external laboratory. The quality of the fiber matched the same quality obtained the previous year when there was no infestation (Fig. 5).

## Discussion

Sarcoptic mange in alpacas is a severe dermatological condition that can sometimes be fatal (McKenna et al. 2005; Borgsteede et al. 2006; Twomey et al. 2009). In our case report, the infestation likely occurred with Mila’s movement to another herd, in which the health status was unknown. Cases have been reported in the literature where the introduction of new individuals already affected in the herd led to outbreaks (Twomey et al. 2009; Lusat et al. 2009). Furthermore, stress from relocation, and changes in diet could be exacerbate ectoparasite infestation.

Treating sarcoptic mange in South American camelids farmed is challenging due to the lack of specific products approved in Europe. Various compounds have been employed to treat sarcoptic mange in alpacas, but their efficacy ranged from low to moderate (Hunter et al. 2004; D’Alterio et al. 2005; Borgsteede et al. 2006; Twomey et al. 2009; Pollock et al. 2017; Beck 2020).

In one study, three alpacas had to be euthanized, one of them died, and the others did not respond to treatment with three doses of doramectin, ivermectin, amitraz, or diazinon (Beck 2020). Repeated subcutaneous therapy with 0.2 mg/kg ivermectin was reported as effective for treating sarcoptic mange in alpacas, but it required at least eight repeated administrations (Twomey et al. 2009). In other studies, topical treatments with doramectin, eprinomectin, or moxidectin had limited success in treating sarcoptic mange in alpacas (Hunter et al. 2004; D’Alterio et al. 2005; Pollock et al. 2017). This could be due to hyperkeratosis, a typical lesion in alpacas affected by sarcoptic mange, which may impede the penetration and absorption of topical compounds (Castilla-Castaño et al. 2021). Another suggested reason for the poor effectiveness of topical acaricides is the absence of lanolin, which can limit their efficacy (Bornstein et al. 2010; Castilla-Castaño et al. 2021).

The topical treatment with amitraz was effective in three *S. scabies*-infected alpacas from the United Kingdom, which were unsuccessfully treated with multiple doses of topical eprinomectin (Lau et al. 2007). In a study by Deak et al. (2021), amitraz was combined with subcutaneous eprinomectin following the use of keratolytics to facilitate the absorption of the topical acaricide. In another study, a combination of topical amitraz and 0.5 mg/kg subcutaneous ivermectin for nine consecutive weeks successfully treated a mixed infestation with sarcoptic and chorioptic mange mites in one alpaca (Castilla-Castaño et al. 2021).

Although the best outcomes have been achieved with the combined use of amitraz and ivermectin, in our case, amitraz was not an option because this compound was unavailable in Italy. Furthermore, all the protocols described as effective with macrocyclic lactones in treating sarcoptic mange required multiple treatments, which consumed a significant amount of time, making these therapeutic protocols challenging to manage.

Fluralaner has delivered excellent results in companion animals (Taenzler et al. 2017; Chiummo et al. 2020; d’Ovidio and Santoro 2021), bighorn sheep (Hering 2020), and poultry (Petersen et al. 2021), effectively and rapidly resolving mite infestations. In a specific study by Hering (2020), bighorn sheep were treated for psoroptic mange with different dosages of fluralaner (oral administration: 5 mg/kg, 25 mg/kg; topical administration: 5 mg/kg, 10 mg/kg). Both oral dosages yielded satisfactory results, while the topical use did not affect mites.

In our study, a single oral administration proved effective in treating mange, leading to the complete recovery of the animal during the 3 months of observation. The present case report, therefore, describes the use of fluralaner at a dosage of 5 mg/kg as effective without the need for repeated treatments, highlighting how treatment with this molecule is more economically sustainable and less time-consuming than other protocols. However, this is a single clinical case, with no existing pharmacodynamics/kinetics studies for using isoxazolines in South American Camelids. Further field trials, especially at the herd level, are required to confirm the efficacy of the treatment protocol employed.

## Data Availability

The materials used during the current study are available from the corresponding author upon reasonable request.
